# Preliminary psychometric linguistic validation of the satisfaction survey for inflatable penile implant in Spanish

**DOI:** 10.1093/sexmed/qfaf089

**Published:** 2025-10-26

**Authors:** Jose M Flores, Bernardita M Ljubetic, Luis F Novaes, Edgardo F Becher, Ignacio Alvarez de Toledo, Alejandro Carvajal, Esteban Calle Correa, Esau Fernandez-Pascual, Juan Ignacio Martinez-Salamanca, Christian Nelson, Carolyn A Salter, Jose Torremade, Maurizio D’Anna, Robert Valenzuela, Alexander Negron, Sergio Moreno, Cristóbal Henríquez, Thomas M Atkinson, John P Mulhall

**Affiliations:** Sexual & Reproductive Medicine, Urology Service, Memorial Sloan Kettering Cancer Center, New York, NY 10065, United States; Sexual & Reproductive Medicine, Urology Service, Memorial Sloan Kettering Cancer Center, New York, NY 10065, United States; Sexual & Reproductive Medicine, Urology Service, Memorial Sloan Kettering Cancer Center, New York, NY 10065, United States; Urology, Universidad de Buenos Aires, Centro de Urologia (CDU), Buenos Aires C1053ABH, Argentina; Urology, Universidad de Buenos Aires, Centro de Urologia (CDU), Buenos Aires C1053ABH, Argentina; Urology, Universidad CES, Medellin, Colombia; Urology, Universidad CES, Medellin, Colombia; Urology, Hospital Universitario La Paz and Lyx Institute of Urology, Universidad Francisco de Vitoria, Madrid 28223, Spain; Urology, Hospital Universitario Puerta de Hierro Majadahonda and Lyx Institute of Urology, Universidad Francisco de Vitoria, Madrid 28223, Spain; Department of Psychiatry and Behavioral Sciences, Memorial Sloan Kettering Cancer Center, New York, NY 10065, United States; Sexual & Reproductive Medicine, Urology Service, Memorial Sloan Kettering Cancer Center, New York, NY 10065, United States; Urology, Hospital Clinic de Barcelona, Barcelona 08036, Spain; Urology, Hospital Clinic de Barcelona, Barcelona 08036, Spain; Urology, Mount Sinai, New York, NY 10029, United States; Urology, Mount Sinai, New York, NY 10029, United States; Urology, Clinica Santa Maria, Universidad Finis Terrae, Santiago 7520378, Chile; Urology, Clinica Santa Maria, Universidad Finis Terrae, Santiago 7520378, Chile; Department of Psychiatry and Behavioral Sciences, Memorial Sloan Kettering Cancer Center, New York, NY 10065, United States; Sexual & Reproductive Medicine, Urology Service, Memorial Sloan Kettering Cancer Center, New York, NY 10065, United States

**Keywords:** erectile dysfunction, penile implant, satisfaction penile implant, linguistic validation

## Abstract

**Background:**

Despite attempts to assess patient satisfaction with inflatable penile prosthesis (IPP) among Spanish-speaking populations, the absence of a validated, purpose-specific questionnaire in Spanish remains a significant gap.

**Aim:**

This study aims to report the preliminary psychometric validation of Spanish Satisfaction Survey for Inflatable Penile Implant (SSIPI).

**Methods:**

Six centers were selected to represent diverse Spanish-speaking populations in Latin America, the United States, and Spain. It included men who had IPP surgery between 6 months and 5 years ago. The translation and cultural adaptation of the SSIPI from English to Spanish followed a systematic multi-staged approach. Each participant received 4 surveys: (i) the final Spanish SSIPI, (ii) the IIEF erectile function domain (IIEF-EFD), (iii) the Self-Esteem and Relationship (SEAR) questionnaire, and (iv) the International Prostate Symptom Score (IPSS). Reliability, internal consistency, and intraclass correlation were evaluated. A convergent analysis was also used to evaluate Spanish SSIPI with SEAR and IIEF-EFD and a divergent analysis between Spanish SSIPI and IPSS.

**Outcomes:**

Spanish SSIPI psychometric validation.

**Results:**

A total of 111 participants were enrolled in the study. The median age was 64. The majority of participants (87%) were partnered, with a median relationship duration of 96 months. The median partner age was 56. Self-reported median time since IPP implantation was 21 months, with individuals indicating that they use the implant with a median of 5 times per month. Inflatable penile prosthesis satisfaction was generally high across all domains. Spanish SSIPI showed good reliability, high consistency, and high internal reliability across all SSIPI domains. Spanish SSIPI domains had significant correlations with the IIEF-EFD and SEAR. Spanish SSIPI domains were not significantly correlated with the overall IPSS score.

**Clinical Implications:**

Spanish SSIPI is a linguistically, culturally, and psychometrically validated patient-reported outcomes measure of satisfaction with IPP, facilitating cross-comparative analysis with previously validated English and French versions.

**Strengths and Limitations:**

This study demonstrates several methodological strengths, including applying a comprehensive psychometric approach, validating across multiple centers, and using a solid methodology to validate Spanish SSIPI. However, there are limitations, particularly the underrepresentation of specific subpopulations and the omission of pre-operative erectile function assessment.

**Conclusion:**

The preliminary Spanish SSIPI demonstrated robust reliability and internal consistency across diverse Spanish-speaking regions.

## Introduction

Erectile dysfunction (ED) is a common condition characterized by a persistent inability to achieve or maintain an erection sufficient for satisfactory sexual activity.^1^ Studies have shown a prevalence of up to 10% in men under 50, increasing to 40% in men aged 60–69 and over 50% in men over 70.^2^ Similar trends of ED are observed for men in Spain and Latin America, increasing with age and with a prevalence between 42% and 53%.^3^^,^^4^ In the United States, the prevalence of ED among Hispanic men has been reported to be up to 20%, increasing with age: 8% in men under 49 years old, 12% between 50 and 59, 40% between 60 and 69, and 54% in men 70 years or older.^5^

From 2001 to 2010, over 53 000 men in the United States underwent penile implant surgery, with 92% receiving an inflatable penile implant (IPP).^6^ Similarly, between 2000 and 2014, 14 114 patients in New York State opted for penile prosthesis surgery, with 88% choosing the IPP.^7^ Conversely, a study from Chile examining records from 2010 to 2020 reported that only 32% of the 1087 men who underwent penile implant surgery chose an IPP.^8^ This variation in prosthesis preference may be attributed to differences in healthcare coverage, as most penile implants in the United States are insurance-funded. In contrast, access to such procedures is more restricted in regions without coverage. The IPP is growing in popularity globally, beyond the United States, due to its enhanced effectiveness and discreet appearance, contributing to its increasing recommendation and promotion.^9^

Assessing patient satisfaction following IPP surgery is critical to determining the procedure’s success. Only 44% of the studies evaluating IPP satisfaction used a validated survey, such as the International Index of Erectile Function (IIEF) or Erectile Dysfunction Inventory of Treatment Satisfaction (EDITS).^10^ However, these questionnaires are inadequate for determining patient satisfaction with the implant because they are designed to assess outcomes in response to erectogenic oral medications and do not address critical aspects related to the prosthesis, such as functionality or discretion. In 2021, the “Satisfaction Survey for Inflatable Penile Implant” (SSIPI) was introduced and validated to assess satisfaction with the IPP.^11^ This tool aimed to provide accurate information about the levels of satisfaction associated with IPP. However, this instrument is currently validated in English and French,^12^ which limits its applicability in Spanish-speaking countries. Despite attempts to assess patient satisfaction with IPP among Spanish-speaking populations, the absence of a validated, purpose-specific questionnaire in Spanish remains a significant gap. Given the regional variations in accent, vocabulary, and cultural context inherent in languages, more than merely translation of the SSIPI questionnaire is required; rigorous linguistic and psychometric validation, defined as the process of establishing the accuracy of a test or assessment, is needed to guarantee accuracy and reliability for comparative outcome analysis. We have previously established the linguistic validation of SSIPI in Spanish.^13^ The purpose of this study is to report the preliminary psychometric validation of Spanish SSIPI.

## Material and methods

### Study population

Each institution secured IRB approval, and our institution authorized an exempt protocol to facilitate the analysis of de-identified data and the execution of psychometric validation. The study was conducted across 6 centers, each strategically selected to represent a wide range of Spanish-speaking populations: Buenos Aires, Argentina, and Santiago, Chile, for South American Spanish speakers; Medellín, Colombia, for South and Central American Spanish speakers; New York City, for US and Central American Spanish speakers; Madrid and Barcelona, for Hispanic Spanish speakers. This final validation step included men who had IPP surgery between 6 months and 5 years ago. Each center provided up to 20 patients. Inclusion criteria were broad, with no exclusions based on surgical complications, marital status, sexual orientation, medical comorbidities, or the specific brand of the prosthesis, to ensure the study findings are generalizable to the broader population of Spanish-speaking IPP patients.

### The Satisfaction Survey for Inflatable Penile Implant

The Satisfaction Survey for Inflatable Penile Implant (SSIPI) was developed and validated in 2021.^11^ It aimed to effectively evaluate patient-reported expectations and satisfaction following IPP surgery. It consists of 16 questions categorized into 4 domains: overall satisfaction (4 questions), pain (2 questions), appearance (6 questions), and function (4 questions). Each question utilized a 5-point Likert scale, with the higher scores indicating more favorable patient outcomes.^11^

### Linguistic validation process in Spanish

The translation and cultural adaptation of the SSIPI from English to Spanish followed a systematic, multi-staged approach designed to preserve the instrument’s conceptual integrity while making it accessible and relevant to Spanish-speaking populations. This approach resulted in the final version used for linguistic validation in this report. The initial process has already been published.^13^ It consisted of 5 steps: (i) forward translation, (ii) reconciliation, (iii) back-translation, (iv) harmonization and review, and (v) cognitive debriefing. Our methodology adhered to best practices established by the International Society for Pharmacoeconomics and Outcomes Research (ISPOR)^14^ and involved forward translation of the 16-item SSIPI into Spanish by 2 independent penile implant surgeons, reconciliation of the 2 Spanish versions into 1, back translation of that version into English by 2 different independent bilingual penile implant surgeons, and comparison to the original SSIPI for conceptual equivalence. The translated SSIPI versions then underwent in-parallel cognitive debriefing at centers located in the United States, Colombia, and Spain. All interviews were in-person or via secure video conference and recorded to determine if misunderstandings or modifications were needed. The cognitive interviews were critical for the validation process, providing qualitative insights into participants’ understanding and interpretation of the questionnaire. The standardized interview protocol was employed to evaluate ease of completion, identify potential issues such as question length or complexity, and gather suggestions for improving the clarity of each question. The feedback obtained during these sessions was meticulously analyzed and incorporated into the final version of the Spanish SSIPI to ensure the highest linguistic and cultural adaptation of the questionnaire for Spanish-speaking populations.

### Questionnaires in Spanish for validation process

Inflatable penile prosthesis patients from the 6 centers received 4 surveys, all of them in Spanish: (i) the final Spanish SSIPI version obtained after the changes suggested during linguistic validation,^13^ (ii) the IIEF erectile function domain (IIEF-EFD), and (iii) the Self-Esteem and Relationship (SEAR) questionnaire, and (iv) the International Prostate Symptom Score (IPSS). The IIEF-EFD,^15^ SEAR,^16^ and IPPS^17^ Questionnaires have been previously validated in Spanish. These questionnaires were selected to replicate meticulously the English validation process into Spanish.^11^ A standardized protocol was implemented to recruit patients with IPP and collect data across all participating centers. Each center included patients who were between 6 months and 5 years post-surgery. They administered various questionnaires, including the Spanish SSIPI, IIEF-EFD, SEAR, and IPSS. Two weeks later, the Spanish SSIPI was re-administered to evaluate test–retest reliability. Patients completed the questionnaires either electronically or in person during follow-up appointments. No compensation was offered to the participants in this study. All collected data were compiled and organized using spreadsheet software and then sent to a central data center for analysis.

### Statistical analysis

Item–total correlations (ie, Pearson *r* coefficient with domain total score) were calculated as a reliability estimate. Pearson *r* values ≥0.40 are indicative of acceptable item–total correlations. Cronbach’s α was calculated for each of the 4 primary domains and the total score at the initial assessment time point as an indicator of internal consistency.^18^ Cronbach’s α values ranging from 0.70 to 0.79 are considered to be fair, whereas values ≥0.80 are indicative of good internal consistency.^19^ Intraclass correlation coefficients (ICCs) were calculated between initial and 14-day follow-up assessments for each of the 4 primary domains and total scores, with values ≥0.70 representing good test–retest reliability.^20^ Convergent validity between the Spanish SSIPI and SEAR, as well as between the Spanish SSIPI and the IIEF-EFD, was assessed using a Pearson *r* coefficient ≥ 0.40 to confirm consistency of the outcomes. In contrast, divergent validity analysis between the SSIPI and the IPSS used a Pearson *r* coefficient ≤ 0.40 to confirm the lack of correlation. Internal structure was analyzed through use of confirmatory factor analysis (CFA). Consistent with the validation of the English SSIPI,^11^ the CFA utilized oblique factors and maximum likelihood estimation and treated the items as continuous variables. Overall CFA fit was determined to be acceptable if 2 of the following were true: comparative fit index (CFI) > 0.90, Tucker–Lewis index (TLI) > 0.90, or root mean square error of approximation (RMSEA) < 0.10.^21^^,^^22^ Analyses were completed using SPSS AMOS version 29.0.2.0.

## Results

Spanish SSIPI is in the supplementary Appendix 1. A total of 111 participants were enrolled in the study. The median age was 64 (IQR 57-72), and 87% were partnered. The median relationship duration was 96 (IQR 32-360) months. The median partner age was 56 (IQR 50-62). Nearly all men were self-identified heterosexual (99%). The comorbidity profile included hypertension (47.7%), hyperlipidemia (25.2%), diabetes (20.7%), coronary artery disease (8.1%), and obstructive sleep apnea (1.8%). The majority (54%) had a history of cancer. Self-reported median time since IPP implantation was 21 (IQR 10-55) months, with individuals indicating that they use the implant with a median of 5 (IQR 3-8) times per month. Participant demographics and clinical characteristics are displayed in Table 1. Mean satisfaction with the IPP is displayed in Table 2 and was generally high across all domains. The mean (SD) overall satisfaction was 4.50 (±0.88), with a mean (SD) for pain, appearance, function, and total scores domains of 4.66 (±0.65), 4.31 (±0.86), 4.61 (±0.64), and 4.44 (±0.74), respectively.

**Table 1:** Participant demographics and clinical characteristics (*N* = 111).

Median age (years, IQR)	64 (57–72)
Partnered (% Yes)	87.4
Median relationship duration (months, IQR)	108 (31–390)
Median partner age (years, IQR)	56 (50–62)
Median time since implant (months, IQR)	21 (10–55)
Still using implant? (% Yes)	97.3
Median number of times using the implant per month (IQR)	5 (3–8)
Sexual orientation (%)	
Straight	99.1
Gay	0.0
Other	0.9
Cancer (%)	
Prostate	49.5
Bladder	1.8
Other	2.7
None	45.9
Comorbidities (%)	
Hypertension	47.7
Hyperlipidemia	25.2
Diabetes	20.7
Coronary artery disease	8.1
Obstructive sleep apnea	1.8
City (%)	
Barcelona, Spain	16.3
Buenos Aires, Argentina	18.0
Madrid, Spain	18.0
Medellín, Colombia	18.0
New York City, United States	18.0
Santiago, Chile	11.7

**Table 2:** The Satisfaction Survey for Inflatable Penile Implant (SSIPI) survey in Spanish unadjusted mean (SD) domains/subscales.

	Mean (SD)
Overall satisfaction	4.50 (±0.88)
Pain	4.66 (±0.65)
Appearance	4.31 (±0.86)
Function	4.61 (±0.64)
Total score	4.44 (±0.74)

### Reliability

Item-total correlations for SSIPI domains are displayed in Table 3 and ranged from 0.53 to 0.92, indicating good reliability of items within each respective domain. Cronbach’s α internal consistencies are shown in Table 4. The consistency was good (ie, ≥0.80) for the overall satisfaction (0.97), pain (0.89), appearance (0.97), function (0.93), and total score (0.98) domains. Table 5 reported the ICC values from initial to 14-day assessments, which ranged from 0.81 to 0.96 across Spanish SSIPI domains, indicating that this tool has good test–retest reliability.

**Table 3:** Item to total correlations for the Satisfaction Survey for Inflatable Penile Implant (SSIPI) survey in Spanish domains.

**Overall satisfaction**	*r*
When you attempted sexual intercourse using the penile implant, how often was it satisfactory for you?¿Cuando mantiene relaciones sexuales utilizando la prótesis de pene, con qué frecuencia le resultan satisfactorias?	0.85
Overall, how satisfied have you been with the penile implant? En general, ¿está usted satisfecho con la prótesis de pene?	0.92
How confident have you been about your ability to engage in sexual activity with the penile implant? ¿Qué nivel de confianza tiene en su capacidad de mantener actividad sexual con la prótesis de pene?	0.89
Have you experienced any regret about having a penile implant placed? ¿Está usted arrepentido de haberse puesto la prótesis de pene?	0.85
**Pain**	
How bothered are you by the pain or discomfort when the implant is activated/inflated? ¿Que grado de molestia experimenta cuando la prótesis esta activada/inflada?	0.89
When the implant is inflated, how often does the pain or discomfort limit your ability to use the implant? Cuando la prótesis está inflada. ¿La molestia o el dolor limita su posibilidad para utilizar la prótesis?	0.89
**Appearance**	
How satisfied are you with the naturalness of your erection with the penile implant when activated/inflated? ¿Está satisfecho con el estado actual de su erección con la prótesis de pene cuando está activada/inflada?	0.84
How satisfied are you with the length of your penis with the penile implant activated/inflated? ¿Está satisfecho con la longitud de su pene cuando la prótesis de pene esta activada/inflada?	0.85
How satisfied are you with the girth/width of your erection with the penile implant when activated/inflated? ¿Está satisfecho con el grosor/ancho de su erección cuando la prótesis de pene está activada/inflada?	0.84
How satisfied are you with the naturalness of your penis when the penile implant is inactivated/deflated? ¿Está satisfecho con la naturalidad de su pene cuando la prótesis de pene está desactivada/desinflada?	0.84
How satisfied are you with your ability to conceal the implant in its inactivated/deflated state? ¿Está satisfecho con la forma de disimular la prótesis de pene en su estado de inactividad/desinflado?	0.76
How satisfied are you with the location of the pump in your scrotum? ¿Está satisfecho con la posición de la bomba en su escroto?	0.68
**Function**	
How often have you had difficulty using the pump in your scrotum to inflate or deflate the device? ¿Con qué frecuencia ha tenido dificultad al utilizar la bomba en su escroto para activar o desactivar el dispositivo?	0.53
How often did the penile implant provide an erection suitable for sexual intercourse? ¿Con qué frecuencia la prótesis de pene le ha proporcionado una erección adecuada para las relaciones sexuales?	0.67
How satisfied are you with the ease of use of your penile implant? ¿Está satisfecho con la facilidad a la hora de usar su prótesis de pene?	0.68
How satisfied are you with the spontaneity of the erection with the penile implant? ¿Está satisfecho con la naturalidad que adquiere su erección con la prótesis de pene?	0.64

Abbreviation: SSIPI, Satisfaction Survey for Inflatable Penile Implant.

**Table 4:** The Satisfaction Survey for Inflatable Penile Implant (SSIPI) survey in Spanish. Cronbach’s α internal consistency statistics.

	# items	Cronbach's α
Overall satisfaction	4	0.97
Pain	2	0.89
Appearance	6	0.97
Function	4	0.93
Total score	16	0.98

**Table 5:** The Satisfaction Survey for Inflatable Penile Implant (SSIPI) survey in Spanish test–retest reliability.

	Intraclass correlation coefficient
Overall satisfaction	0.94
Pain	0.81
Appearance	0.94
Function	0.86
Total score	0.96

### Validity

Convergent and divergent validity values are displayed in Table 6. Spanish SSIPI total scores had a 0.53 correlation with the SEAR total score (*P* < .001), as an indicator of convergent validity. With the exception of the relationship between the SEAR Self Esteem and Spanish SSIPI Pain domain (*r* = 0.18, *P* = .055) and between the SEAR Confidence and Spanish SSIPI Pain Domain (*r* = 0.24, *P* = .013), the remaining SEAR and Spanish SSIPI coefficients ranged from 0.25 to 0.59 (*p*s < .01). Spanish SSIPI domains had moderate but significant correlations with the IIEF-EFD (*r* range 0.31-0.42, *p*s < .001). With respect to divergent validity, as expected, Spanish SSIPI domains were not significantly correlated with the overall IPSS score (*r* range -0.05 to -0.11, *P* range 0.26-0.57). The CFA fit was determined to be acceptable, as CFI = 0.93 and TLI = 0.90. The RMSEA value was 0.104.

**Table 6:** The Satisfaction Survey for Inflatable Penile Implant (SSIPI) survey in Spanish convergent and discriminant validity (*r*, *P*-value).

	Spanish SSIPI domain				
Legacy measure	Overall satisfaction	Pain	Appearance	Function	Total score
Convergent validity					
SEAR Sexual Relationship	0.59 (<0.001)	0.30 (<0.001)	0.40 (<0.001)	0.56 (<0.001)	0.55 (<0.001)
SEAR Self-Esteem	0.42 (<0.001)	0.18 (0.055)	0.32 (<0.001)	0.38 (<0.001)	0.38 (<0.001)
SEAR Confidence	0.51 (<0.001)	0.24 (0.013)	0.36 (<0.001)	0.45 (<0.001)	0.47 (<0.001)
SEAR Overall Relationship	0.49 (<0.001)	0.25 (0.009)	0.32 (<0.001)	0.43 (<0.001)	0.46 (<0.001)
SEAR Total	0.57 (<0.001)	0.29 (0.002)	0.40 (<0.001)	0.54 (<0.001)	0.53 (<0.001)
IIEF—Erectile Function	0.35 (<0.001)	0.33 (<0.001)	0.42 (<0.001)	0.31 (<0.001)	0.41 (<0.001)
Divergent validity					
IPSS	-0.06 (0.508)	-0.11 (0.256)	-0.05 (0.574)	-0.11 (0.275)	-0.08 (0.387)

Abbreviations: SSIPI, Satisfaction Survey for Inflatable Penile Implant; SEAR, Self-Esteem and Relationship Questionnaire; IIEF, International Index of Erectile Function; IPSS, International Prostate Symptom Score.

## Discussion

When linguistically and culturally validating a patient-reported outcomes questionnaire, it is important to address various forms of equivalence, such as semantic, conceptual, operational, psychometric, and criterion equivalence.^14^ These forms of equivalence ensure that the translated questionnaire is a linguistic match and retains its validity and reliability in the new context. We are reporting the preliminary first Spanish-validated linguistic survey to evaluate patient satisfaction with IPP for Spanish speakers. We also focus on educating health providers regarding the complex process of validating a survey in a selected language. Linguistic validation is a long process that includes forward translation, reconciliation, back translation, comparison to the original survey, and cognitive debriefing. The cognitive debriefing is vital to evaluate the ease of completion, identify potential issues, and gather suggestions that are incorporated into the final version, which is used with other surveys already validated in the language selected to assess correlations, internal consistency, and convergent and divergent validities, which are the final steps to validate the survey linguistically. As we demonstrated in this study, it is not enough to translate it; it is critical to consider the regional variations in accent, vocabulary, and cultural context inherent in languages and take the proper steps for linguistic validation of any survey to guarantee accuracy and reliability for comparative outcome analysis for research and patient-reported outcomes. Validated questionnaires are pivotal in clinical trials and research studies, where data accuracy is critical. A poorly validated translation can compromise the comparability of results between populations. The Spanish SSIPI version follows the stringent guidelines set by the International Society for Pharmacoeconomics and Health Outcomes (ISPOR),^14^ a gold standard for validation processes, ensuring that the instrument’s results are comparable across different linguistic and cultural groups. The final step of the process involves completing the psychometric validation to confirm the questionnaire’s suitability for use in Spanish-speaking populations.^14^ In the present study, the preliminary Spanish SSIPI demonstrated robust reliability and internal consistency across diverse regions in Spain, the United States, and Latin America. It was linguistically and culturally validated and is available to assess patient-reported outcomes regarding satisfaction with IPP, facilitating cross-comparative analysis with previously validated English and French versions.

Psychometric validation of the Spanish SSIPI may address important existing gaps in evaluating patient-reported outcomes related to IPP surgery. Chouhan et al.^23^ conducted a systematic review analyzing the most cited articles between 2009 and 2019 regarding IPP surgical outcomes. The review revealed a need for more high-quality evidence, as many studies exhibited significant methodological limitations, including the absence of validated tools to assess patient satisfaction or the reliance on non-standardized assessment measures. Although Caraceni et al.^24^ developed the “Quality of Life and Sexuality with Penile Prostheses” questionnaire to evaluate IPP satisfaction, 2 key limitations persist with this questionnaire: Its validation is restricted to the Italian language, limiting its broader applicability, and it does not include critical aspects of IPP, such as aesthetic or functional concerns. Additionally, the inclusion of non–prosthesis-related elements, such as sexual frequency, further detracts from the questionnaire’s specific focus on IPP outcomes. More literature needs to address IPP satisfaction within the Spanish-speaking population, and regrettably, the extant studies predominantly rely on surgeon-administered surveys or the EDITS questionnaire. Recently, Garcia et al.^25^ published the satisfaction rate with IPP in men with and without Peyronie’s disease in a Spanish-speaking population (Spain). The authors reported that 86% of the patients were very or moderately satisfied; however, this study used a surgeon-administered non-validated survey.

This study demonstrates several methodological strengths, including applying a comprehensive psychometric approach, validation across multiple centers, and rigorous methodology to validate SSIPI in Spanish. Also, a key part of the study was that it was independent of industry sponsorship, minimizing potential bias. However, there are limitations, particularly the underrepresentation of specific subpopulations, such as gay men and men with Peyronie’s disease; the omission of pre-operative erectile function assessment may have influenced post-operative satisfaction outcomes. Another significant limitation of the study is its small sample size, which could undermine statistical power, reduce replicability, and compromise the robustness of psychometric evaluations presented in this study. With only 111 participants for a 16-item measure, the CFA results may be biased. Additionally, future steps would be needed to compare multiple groups across age, region, or usage patterns. This limitation also affects bivariate tests, preventing the validation of the measure across different Spanish-speaking regions and raising concerns about potential cultural specificity and, potentially, the generalizability of the instrument across diverse Spanish-speaking populations could be limited, which we attempted to minimize by presenting data from 3 different regions (South America, Spain, and the United States). Additionally, the exclusion of reservoir-related questions and the need for partner satisfaction measures represent further limitations of this study that should be included in future research.

## Conclusion

The preliminary Spanish SSIPI version demonstrated robust reliability and internal consistency across diverse Spanish-speaking regions, including Spain, the United States, and Latin America. The Spanish SSIPI underwent comprehensive linguistic and cultural validation, which could be used by sexual medicine physicians performing IPP surgeries to gather patient-reported outcomes on satisfaction with IPP in Spanish-speaking patients.

## Supplementary Material

SSIPI_LV_Phase_2_Supplementary_17_qfaf089
