# Supplementary material for: Preliminary psychometric linguistic validation of the satisfaction survey for inflatable penile implant in Spanish
Source: Sex Med. 2025 Oct 26;13(5):qfaf089. doi: 10.1093/sexmed/qfaf089 (PMC12554161; doi:10.1093/sexmed/qfaf089)
Supplement: SSIPI_LV_Phase_2_Supplementary_17_qfaf089 [file ssipi_lv_phase_2_supplementary_17_qfaf089.docx]

The Satisfaction Survey for Inflatable Penile Implant (SSIPI) survey in Spanish

ENCUESTA DE SATISFACCIÓN SOBRE EL USO DE LA PRÓTESIS INFLABLE DE PENE

Por favor, rogamos que responda a las siguientes preguntas basándose en su experiencia con su prótesis de pene.

**1. ¿Cuando mantiene relaciones sexuales utilizando la prótesis de pene, con qué frecuencia le resultan satisfactorias?**

1. Casi Nunca o nunca

2. Pocas veces (mucho menos de la mitad de las veces)

3. Algunas veces (aproximadamente la mitad de las veces)

4. Muchas veces (mucho más de la mitad de las veces)

5. Casi siempre o siempre

**2. En general, ¿está usted satisfecho con la prótesis de pene?**

1. Muy insatisfecho

2. Algo insatisfecho

3. Ni satisfecho ni insatisfecho

4. Algo satisfecho

5. Muy satisfecho

**3. ¿Qué nivel de confianza tiene en su capacidad de mantener actividad sexual con la prótesis de pene?**

1. Completamente inseguro

2. Algo inseguro

3. Ni seguro ni inseguro

4. Algo seguro

5. Muy seguro

**4- ¿Está usted arrepentido de haberse puesto la prótesis de pene?**

1. Muy arrepentido

2. Bastante arrepentido

3. Algo arrepentido

4. Un poco arrepentido

5. Nada arrepentido

**5. ¿Que grado de molestia experimenta cuando la prótesis esta activada/inflada?**

1. Muchísimo

2. Bastante

3. Algo

4. Un poco

5. Ninguna

**6-Cuando la prótesis está inflada. ¿La molestia o el dolor limita su posibilidad para utilizar la prótesis?**

1. Casi siempre-siempre

2. Muchas veces (mucho más de la mitad de las veces)

3. Algunas veces (casi la mitad de las veces)

4. Pocas veces (mucho menos que la mitad de las veces)

5. Casi nunca-nunca

**7. ¿Está satisfecho con el estado actual de su erección con la prótesis de pene cuando está activada/inflada?**

1. Muy insatisfecho

2. Algo insatisfecho

3. Ni satisfecho ni insatisfecho

4. Algo satisfecho

5. Muy satisfecho

**8. ¿Está satisfecho con la longitud de su pene cuando la prótesis de pene esta activada/inflada?**

1. Muy insatisfecho

2. Algo insatisfecho

3. Ni satisfecho ni insatisfecho

4. Algo satisfecho

5. Muy satisfecho

**9. ¿Está satisfecho con el grosor/ancho de su erección cuando la prótesis de pene está activada/inflada?**

1. Muy insatisfecho

2. Algo insatisfecho

3. Ni satisfecho ni insatisfecho

4. Algo satisfecho

5. Muy satisfecho

**10. ¿Está satisfecho con la naturalidad de su pene cuando la prótesis de pene está desactivada/desinflada?**

1. Muy insatisfecho

2. Algo insatisfecho

3. Ni satisfecho ni insatisfecho

4. Algo satisfecho

5. Muy satisfecho

**11- ¿Está satisfecho con la forma de disimular la prótesis de pene en su estado de inactividad/desinflado?**

1. Muy insatisfecho

2. Algo insatisfecho

3. Ni satisfecho ni insatisfecho

4. Algo satisfecho

5. Muy satisfecho

**12. ¿Está satisfecho con la posición de la bomba en su escroto?**

1. Muy insatisfecho

2. Algo insatisfecho

3. Ni satisfecho ni insatisfecho

4. Algo satisfecho

5. Muy satisfecho

**13. ¿Con qué frecuencia ha tenido dificultad al utilizar la bomba en su escroto para activar o desactivar el dispositivo?**

1. Casi siempre o siempre

2. Muchas veces (mucho más de la mitad de las veces)

3. Algunas veces (aproximadamente la mitad de las veces)

4. Pocas veces (mucho menos de la mitad de las veces)

5. Casi Nunca o nunca

**14. ¿Con qué frecuencia la prótesis de pene le ha proporcionado una erección adecuada para las relaciones sexuales?**

1. Casi Nunca o nunca

2. Pocas veces (mucho menos de la mitad de las veces)

3. Algunas veces (aproximadamente la mitad de las veces)

4. Muchas veces (mucho más de la mitad de las veces)

5. Casi siempre o siempre

**15. ¿Está satisfecho con la facilidad a la hora de usar su prótesis de pene?**

1. Muy insatisfecho

2. Algo insatisfecho

3. Ni satisfecho ni insatisfecho

4. Algo satisfecho

5. Muy satisfecho

**16. ¿Está satisfecho con la naturalidad que adquiere su erección con la prótesis de pene?**

1. Muy insatisfecho

2. Algo insatisfecho

3. Ni satisfecho ni insatisfecho

4. Algo satisfecho

5. Muy satisfecho

Puntuación Total:

Valoración:

Satisfacción global 1-4:

Dolor 5-6:

Apariencia 7-12:

Función 13-16:
